# Overexpression of LncRNA GHET1 predicts an unfavourable survival and clinical parameters of patients in various cancers

**DOI:** 10.1111/jcmm.14486

**Published:** 2019-06-28

**Authors:** Yao‐Fei Jiang, Hong‐Yan Zhang, Jin Ke, Hui Shen, Hai‐Bin Ou, Yu Liu

**Affiliations:** ^1^ Hubei Cancer Clinical Study Centre & Hubei Key Laboratory of Tumor Biological Behaviors Zhongnan Hospital, Wuhan University Wuhan China; ^2^ Department of Radiation and Medical Oncology Zhongnan Hospital, Wuhan University Wuhan China; ^3^ Department of Oral and Maxillofacial Surgery, School and Hospital of Stomatology Wuhan University Wuhan China

**Keywords:** cancers, clinical Parameters, GHET1, LncRNA, prognosis

## Abstract

Recently, increasing studies have reported that long non‐coding RNA (lncRNA) gastric carcinoma highly expressed transcript 1 (GHET1) is highly expressed in variety of cancers and relevant to poor prognosis of cancer patients. Nevertheless, the results were inconsistent and the systematic analysis of lncRNA GHET1 in cancers has not been inspected. Thus, we aim to evaluate the relationship between lncRNA GHET1 expression and clinical outcomes in human cancers. We searched keywords in PubMed, Web of Science, Embase, Cochrane Library and ClinicalTrial.gov. Stata SE12.0 software was used in the quantitative meta‐analysis. Pooled hazard ratio (HR) and odds ratio with 95% confidence interval (95% Cl) were calculated to evaluate the clinical significance of lncRNA GHET1. Twelve studies totalling 761 patients with cancers were included for analysis. The pooled results of this study indicated that high lncRNA GHET1 expression level was significantly associated with poor overall survival (OS, HR = 2.30, 95% CI: 1.75‐3.02) in human cancers. The statistical significance was also detected in subgroup analysis stratified by analysis method, cancer type, sample size and follow‐up time respectively. In addition, the elevated lncRNA GHET1 expression was also significantly related to more advanced clinical stage, earlier lymph node metastasis, earlier distant metastasis and bigger tumour size. LncRNA GHET1 may serve as a promising biomarker for prognosis in Asians with cancers.

## 1 INTRODUCTION

As a leading cause of death worldwide, cancer is a big problem for human beings.^1^ It was reported that there were 14.1 million newly diagnosed cases and 8.2 million deaths resulted from poor prognosis and post‐operative care each year despite of advancement of cancer diagnosis and treatment.^1^, ^2^, ^3^ Therefore, novel biomarkers for predicting cancer prognosis were important and urgently needed for therapeutic decision‐making and improving the care after surgery.^4^

Long non‐coding RNAs (lncRNAs), longer than 200 nucleotides in length, are mRNA‐like transcripts and cannot be translated into proteins.^5^, ^6^ It is being increasingly recognized that abnormal expression of lncRNAs is specifically related to tumorigenesis, tumour progression and metastasis.^7^, ^8^ As a kind of potential and novel cancer biomarker, there were more and more lncRNAs found every year. Although some lncRNAs functioned as protective genes, such as ANRIL, GAS5, MEG3,^9^, ^10^, ^11^ most act as oncogenes, such as HOTAIR, RMRP, CCAT2 and TUG1.^12^, ^13^, ^14^, ^15^ Therefore, although IncRNAs were known as the noise of transcriptions initially, increasing attentions were attached to them because of their important roles in development of diseases and diverse biological processes, and they may serve as promising biomarkers in predicting prognosis of cancer.^14^, ^15^, ^16^

Gastric carcinoma highly expressed transcript 1 (GHET1), has been identified as an lncRNA recently. It has been confirmed to be an oncogene in a variety of cancers, including the hepatocellular carcinoma, gastric cancer and colorectal cancer. LncRNA GHET1 activated by H3K27 acetylation promotes cell tumorigenesis through regulating ATF1 in the hepatocellular carcinoma.^17^ Knockdown of lncRNA GHET1 inhibits cell‐cycle progression and invasion.^18^ Knockdown of lncRNA GHET1 inhibits cell proliferation and invasion of the colorectal cancer.^19^ Overexpression of lncRNA GHET1 promotes the development of multidrug resistance in the gastric cancer cells.^20^

Despite some studies that reported the association between lncRNA GHET1 and cancers, there has been still no consistent conclusion on the prognostic value of lncRNA GHET1 in cancer patients because of different outcomes and limited sample size in each individual study.^17^, ^18^, ^19^, ^20^, ^21^, ^22^ Therefore, we conducted this meta‐analysis to identify the relationship between the expressions of lncRNA GHET1 in a variety of human cancers and the patients’ overall survival (OS) as well as other clinical parameters, identifying the prognostic value of lncRNA GHET1 as a novel biomarker for human cancers.

## 2 MATERIALS AND METHODS

Our systematic review and meta‐analysis was reported according to the recommendations of the PRISMA statement.^23^

### 2.1 Literature retrieval strategy

In order to collect all articles eligible for this study, we retrieved keywords in different databases, including PubMed, Embase, Web of Science, Cochrane Library and ClinicalTrials.gov from their inception to 26 November 2018. Keywords and MeSH terms were used in combination as follows: (‘GHET1 transcript’ OR ‘gastric carcinoma high expressed transcript 1, human’ OR ‘lncRNA‐GHET1, human’ OR ‘gastric carcinoma proliferation enhancing transcript 1, human’) AND (‘cancer’ OR ‘tumour’ OR ‘neoplasm’ OR ‘carcinoma’) AND (‘pathology’ OR ‘pathological feature’ OR ‘prognosis’ OR ‘clinical outcome’ OR ‘survival’). Other additional studies were obtained by screening the reference list. The articles only written in English were included in this meta‐analysis.

### 2.2 Inclusion and exclusion criteria

Eligible articles enrolled in this meta‐analysis meet the following criteria: (a) patients included were diagnosed with cancers; (b) the expression of lncRNA GHET1 in tissue specimens was reported; (c) the associations between lncRNA GHET1 expression and prognosis or clinical parameters of cancers were reported; (d) sufficient data were eligible to calculate the hazard ratio (HR) with 95% confidence intervals (CI) for OS, or odds ratios (ORs) with 95% CI for clinical parameter. (e) Language was used in English. Exclusion criteria were (included): (a) studies without available data for survival and clinical parameters; (b) overlapping data; or (c) letters, reviews, case reports and expert opinions.

### 2.3 Data extraction and quality assessment

Two authors extracted the information and data from included studies independently, and any disagreement was resolved by consulting with the third author. The information and data were as following: family name of first author, year of publication, cancer type, total sample size, tumour stage, sample type, cut‐off value of lncRNA GHET1, detection method, outcome measures, follow‐up time and type of analysis method. Meanwhile, some other clinical parameters were recorded, including gender, age, lymph node metastasis, TNM stage, differentiation, distant metastasis, tumour size and smoke history. For the HRs and 95% CIs of survival, the data were recorded directly from articles provided the detail information. Two individual authors extracted the data from those provided Kaplan‐Meier curves only with the Engauge Digitizer version 4.1. To evaluate the quality of included studies, the Newcastle‐Ottawa Scale (NOS) was applied, whose score is ranging from 0 to 9 points.^24^, ^25^ Studies’ NOS score, 7‐9, has been regarded as high quality.

### 2.4 Statistical methods

Stata SE12.0 software (StataCorp LLC, College Station, Texas) was used to calculate the statistical analysis of HRs for OS and ORs for clinical parameters. Fixed‐effects model was used to pool data with statistical heterogeneity determined by the inconsistency index (*I* ^2^ ≥ 50%) and the chi‐squared test (*P* ≤ 0.10). If the statistical heterogeneity was significant, random‐effects model was then preformed.^26^, ^27^, ^28^, ^29^ In addition, we preformed Begg's rank correlation test to assess the publication bias for the HRs for OS, determined as positive by Pr>|z|≤0.1. We also did a sensitivity analysis to check the stability of results for OS. Moreover, subgroup analyses of OS were performed.

## 3 RESULTS

### 3.1 Literature search

**Figure 1:**
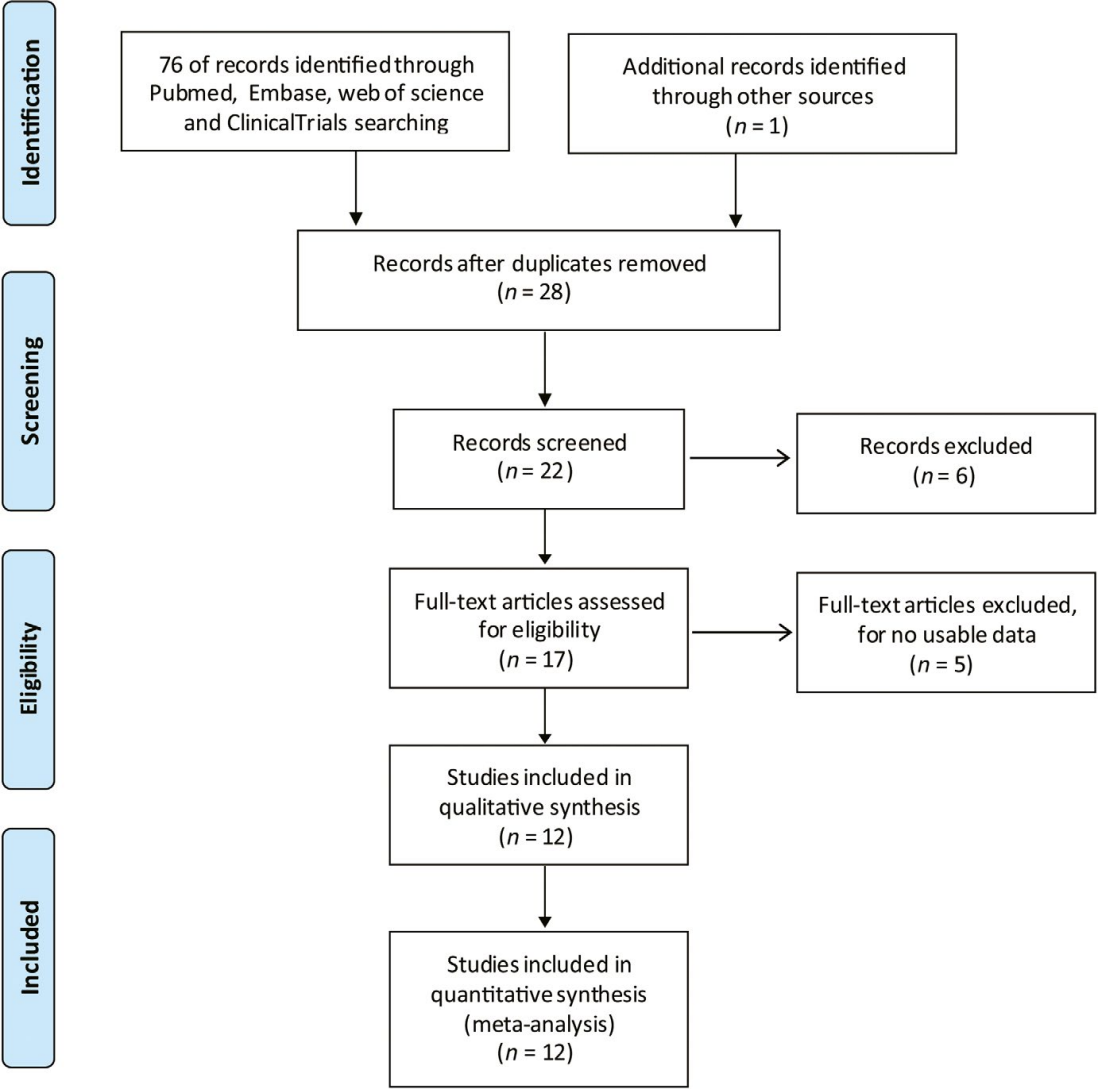
Flow diagram of the literature retrieval and selection

A total of 12 eligible articles containing 761 patients with cancers were included in this meta‐analysis after screening titles, abstracts or full texts.^18^, ^21^, ^22^, ^30^, ^31^, ^32^, ^33^, ^34^, ^35^, ^36^, ^37^, ^38^ The process of article selection was shown in Figure 1.

### 3.2 Main information of included studies

In these studies, the sample size ranged from 42 to 105, among a total of 761 participants. The main characteristics of included studies were presented in Table 1. Among the 12 studies, there were various types of cancers, including non‐small cell lung cancer (two articles), hepatocellular carcinoma, bladder cancer, oesophageal squamous cell carcinoma, head and neck cancer, breast cancer (two articles), gastric cancer (three articles) and pancreatic cancer. Among these studies, one came from Iran, all others came from China. The publication period of these articles ranged from 2014 to 2018. The samples of all these studies were from cancer and matched normal tissues. In addition, the level of lncRNA GHET1 was detected by quantitative reverse transcription polymerase chain reaction (qRT‐PCR). There were four different cut‐off points for the 12 studies: four median level, four median ratio, one average level and three not reported. In regard to disease outcomes, eight studies reported OS, one study reported DFS, the remnant reported clinical parameters. The high‐quality studies in this meta‐analysis have NOS score: 7‐9.

**Table 1:** The main information of included studies in the meta‐analysis

References	Country	Cancer type	Samples （n）	Tumour stage	Sample type	Cut‐off value	Detection method	Outcome measure	Follow‐up	Analysis method	NOS
Xia et al^18^	China	Gastric cancer	42	I‐IV	Cancer and matched normal tissues	Median level	qRT‐PCR	CP	NR	NR	6
Jin et al^21^	China	Hepatocellular carcinoma	68	I‐IV	Cancer and matched normal tissues	Median level	qRT‐PCR	OS	5 y	Kaplan‐Meier	9
Guan et al^22^	China	Non‐small cell lung cancer	52	I‐III	Cancer and matched normal tissues	NR	qRT‐PCR	OS	55 mo	Multivariate	8
Li et al^30^	China	Bladder cancer	80	NR	Cancer and matched normal tissues	Median ratio	qRT‐PCR	OS	5 y	Kaplan‐Meier	8
Liu et al^31^	China	Oesophageal squamous cell carcinoma	55	I‐IV	Cancer and matched normal tissues	Median ratio	qRT‐PCR	CP	NR	NR	6
Liu & Wu^32^	China	Head and neck cancer	86	I‐III	Cancer and matched normal tissues	Median ratio	qRT‐PCR	OS	>5	Kaplan‐Meier	8
Sarrafzadeh et al^33^	Iran	Breast cancer	47	O‐IV	Cancer and matched normal tissues	Average level	qRT‐PCR	CP	NR	NR	7
Shen et al^34^	China	Non‐small cell lung cancer	105	I‐IV	Cancer and matched normal tissues	Median ratio	qRT‐PCR	OS	>5 y	Kaplan‐Meier	9
Song et al^35^	China	Breast cancer	60	I‐IV	Cancer and matched normal tissues	Median level	qRT‐PCR	OS	5 y	Kaplan‐Meier	8
Yang et al^36^	China	Gastric cancer	42	I‐IV	Cancer and matched normal tissues	NR	qRT‐PCR	OS	40 mo	Kaplan‐Meier	7
Zhou et al^37^	China	Pancreatic cancer	64	I‐IV	Cancer and matched normal tissues	Median level	qRT‐PCR	CP	NR	NR	7
Yang et al^38^	China	Osteosarcoma	60	I‐IV	Cancer and matched normal tissues	NR	qRT‐PCR	OS	5 y	Kaplan‐Meier	7

Abbreviations: CP, clinical parameter; NR, not reported; OS, overall survival; qRT‐PCR, quantitative reverse‐transcriptase polymerase chain reaction.

### 3.3 Association between lncRNA GHET1 expression and OS

**Figure 2:**
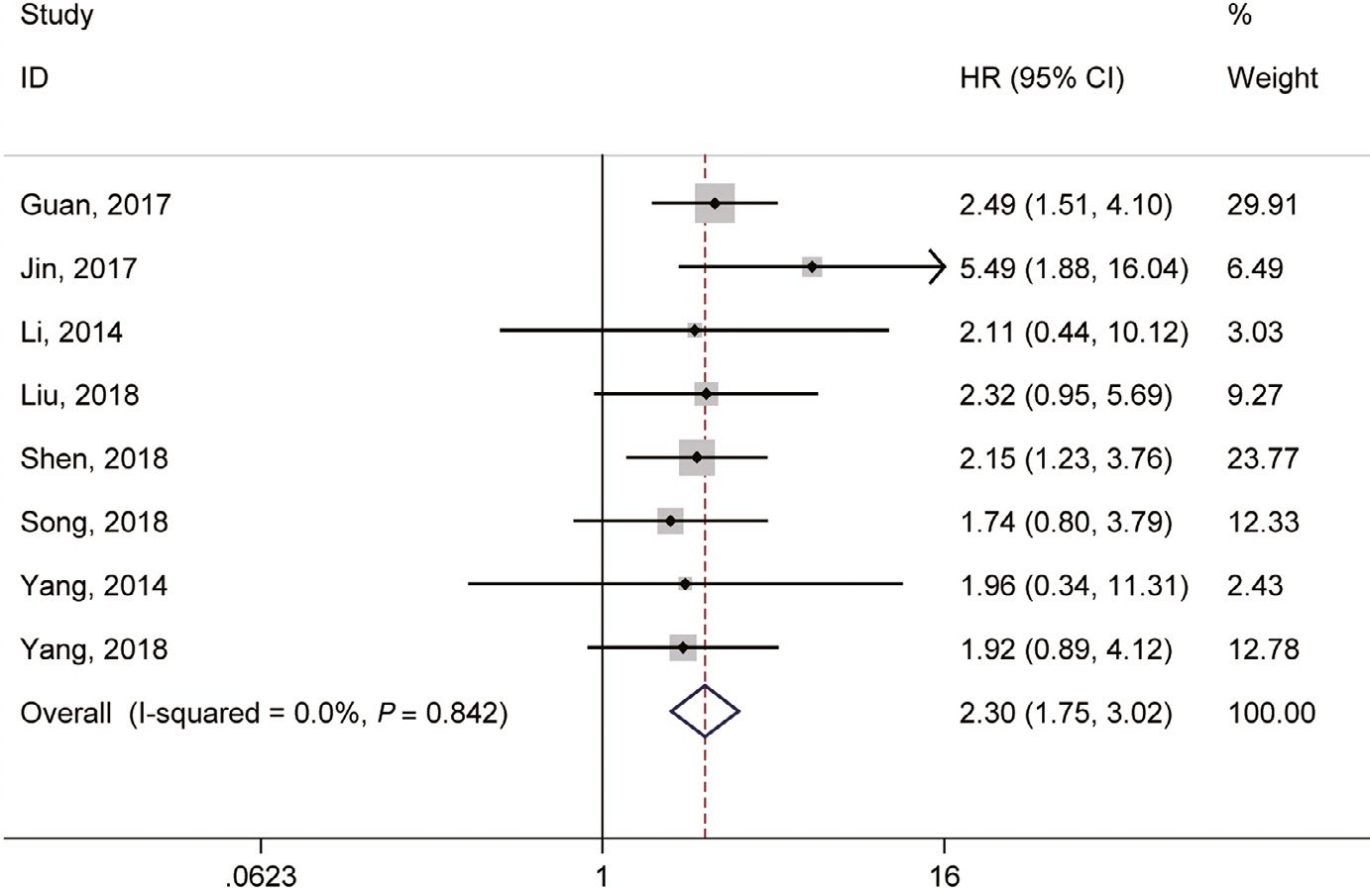
Meta‐analysis of the pooled hazard ratios (HRs) of overall survival in patients with cancer

**Figure 3:**
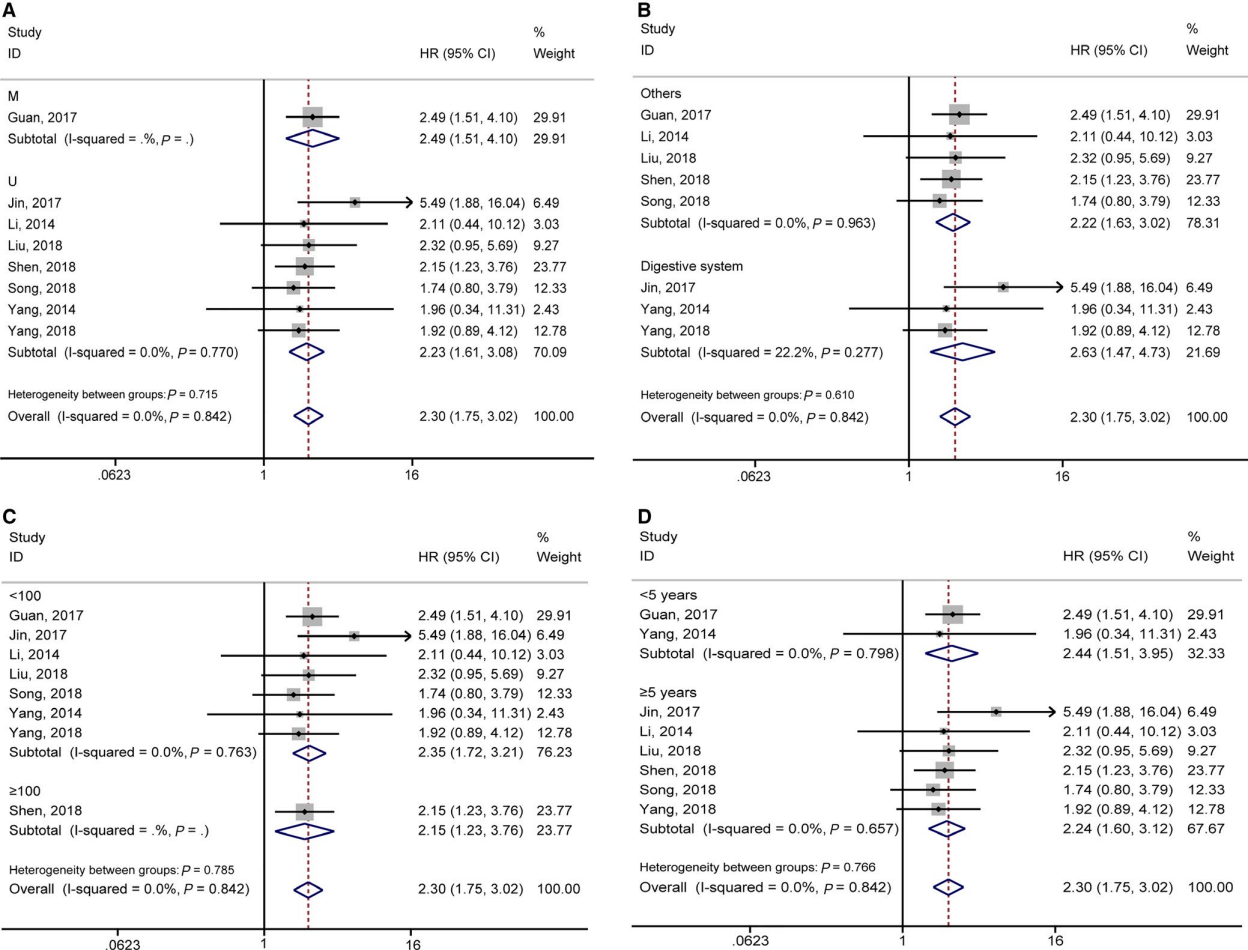
Forest plots of hazard ratios (HRs) for the relationship between high Musashi‐1 expression and overall survival (OS): (A) stratified by analysis type; (B) stratified by cancer type; (C) stratified by sample size; (D) stratified by follow‐up time

Seven articles investigated the association between lncRNA GHET1 expression and OS with a total of 553 cancer patients.^21^, ^22^, ^30^, ^32^, ^34^, ^35^, ^36^, ^38^ We used fix‐effect model to analysis the HR of OS because of no significant heterogeneity among studies (*I* ^2^ = 0.0%, *P* = 0.842; Figure 2). Meta‐analysis of these studies revealed that high lncRNA GHET1 expression level was significantly relative to poor OS in human cancer (HR: 2.30, 95% CI: 1.75‐3.02). Then we performed subgroup meta‐analysis, which was stratified by analysis type, cancer type, sample size and follow‐up time (Table 2). We found that high lncRNA GHET1 expression level was positively associated with shorter OS in studies using multivariate analysis method (HR: 2.49, 95% CI: 1.51‐4.10) and non‐multivariate (HR: 2.23, 95% CI: 1.61‐3.08). In addition, significant association was also found in subgroup meta‐analysis stratified by cancer type. The HRs for the high lncRNA GHET1 expression group versus the low lncRNA GHET1 expression group were 2.63 (95% CI: 1.47‐4.73) in digestive system cancer and 2.22 (95% CI: 1.63‐3.02) in other cancers. After stratified by sample size, the HRs were 2.35 (95% CI: 1.72‐3.21) in studies containing the number of patients <100 and 2.15 (95% CI: 1.23‐3.76) in studies with the number of patients ≥100. After stratification by follow‐up time, the HRs were 2.24 (95% CI: 1.60‐3.12) in studies with follow‐up time ≥5 years and 2.44 (95% CI: 1.51‐3.95) when follow‐up time was <5 years (Figure 3).

**Table 2:** The results of subgroup analyses of OS

Variables		Studies (n)	Number of patients (n)	HR	95% CI	*I^2^* (%)	*P*_h_	Model
Analysis type	Multivariate	1	52	2.49	1.51‐4.10	—	—	Fixed
	Non‐multivariate	7	501	2.23	1.61‐3.08	0	0.770	Fixed
Cancer type	Digestive system	3	170	2.63	1.47‐4.73	22.2	0.277	Fixed
	Others	5	383	2.22	1.63‐3.02	0	0.963	Fixed
Sample size	<100	7	448	2.35	1.72‐3.21	0	0.763	Fixed
	≥100	1	105	2.15	1.23‐3.76	—	—	Fixed
Follow‐up time	≥5 y	6	459	2.24	1.60‐3.12	0	0.657	Fixed
	<5 y	2	94	2.44	1.51‐3.95	0	0.798	Fixed

Abbreviation: CI, confidence interval; HR, hazard ratios; OS, overall survival.

### 3.4 Association between lncRNA GHET1 expression and clinical parameters

**Figure 4:**
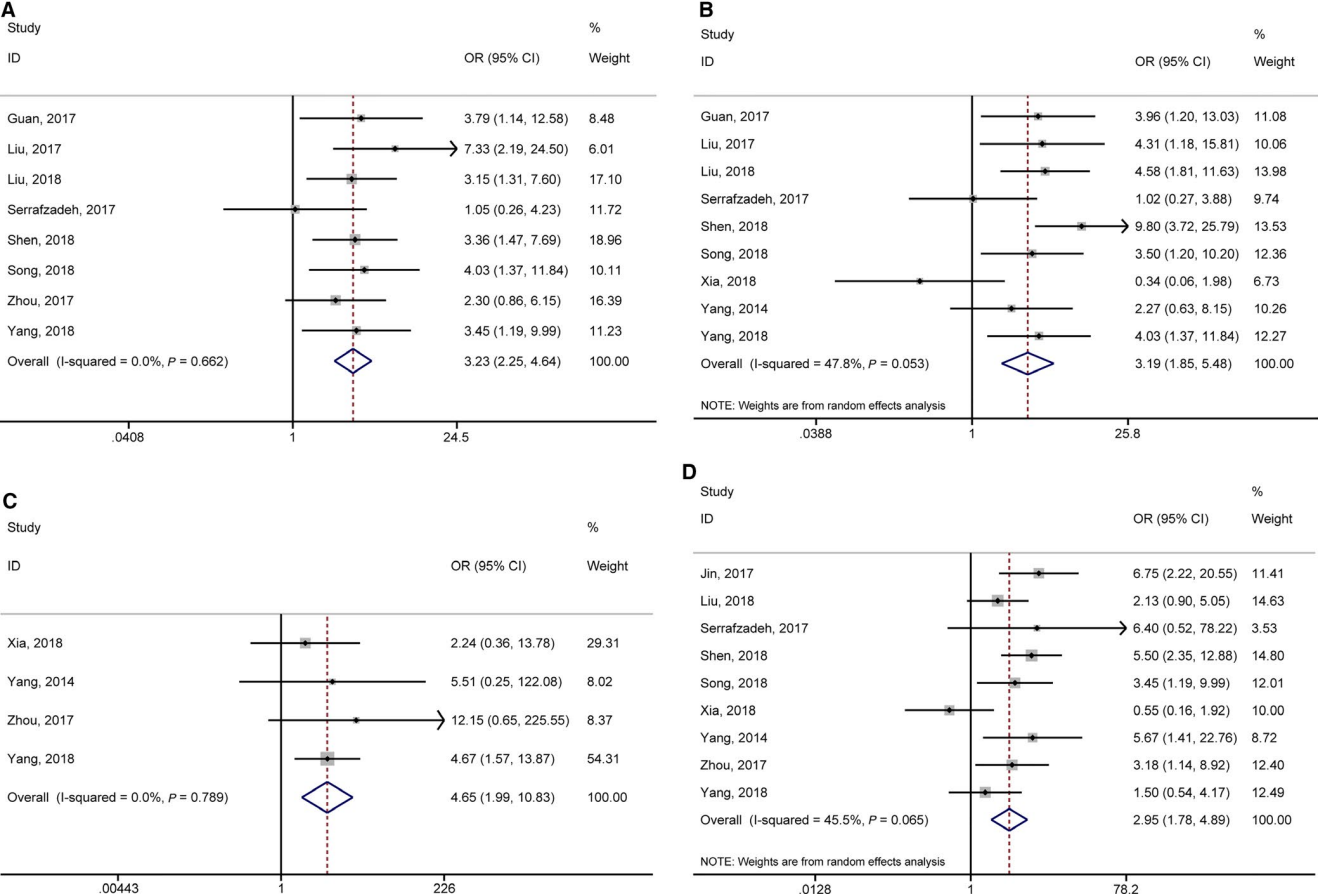
Forest plots of odds ratios (ORs) for the relationship between high Musashi‐1 expression and clinical parameters: (A) TNM stage; (B) lymph node metastasis; (C) distant metastasis; (D) tumour size

**Figure 5:**
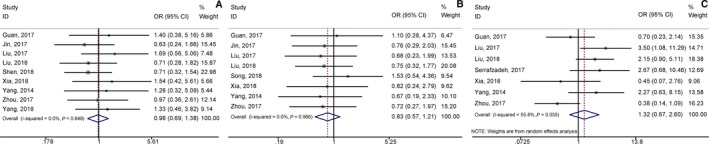
Forest plots of odds ratios (ORs) for the relationship between high Musashi‐1 expression and clinical parameters: (A) gender; (B) age; (C) differentiation

According to meta‐analysis results of Table 3, we found that the high expression of lncRNA GHET1 was associated with more advanced clinical stage (OR = 3.23, 95% CI: 2.25‐4.64), earlier lymph node metastasis (OR = 3.19, 95% CI: 1.85‐5.48), earlier distant metastasis (OR = 4.65, 95% CI: 1.99‐10.83) and bigger tumour size (OR = 2.95, 95% CI: 1.78‐4.89) (Figure 4). However, no statistical significance was found in gender (OR = 0.98, 95% CI: 0.69‐1.38), age (OR = 0.83, 95% CI:0.57‐1.21), differentiation (OR = 1.32, 95% CI: 0.67‐2.60) (Figure 5).

**Table 3:** Meta‐analysis of the relationship between over‐expressed LncRNA GHET1 and clinical parameters

Categories	Studies (n)	Number of patients (n)	OR	95% CI	Heterogeneity		Model
					*I^2^* (%)	*P*_h_	
Gender (male versus female)	9	574	0.98	0.69‐1.38	0	0.848	Fixed
Age (>60 vs <60 y)	8	469	0.83	0.57‐1.21	0	0.966	Fixed
TNM stage (III/IV vs I/II)	8	529	3.23	2.25‐4.64	0	0.662	Fixed
LNM (yes vs no)	9	549	3.19	1.85‐5.48	47.8	0.053	Random
Distant metastasis (yes vs no)	4	208	4.65	1.99‐10.83	0	0.789	Fixed
Differentiation (poor vs good)	7	388	1.32	0.67‐2.60	55.8	0.035	Random
Tumour size (bigger vs smaller)	8	574	2.95	1.78‐4.89	45.5	0.065	Random

Abbreviations: CI, confidence interval; lncRNA, long non‐coding RNA; LNM, lymph node metastasis; OR, odds ratio.

### 3.5 Publication bias

**Figure 6:**
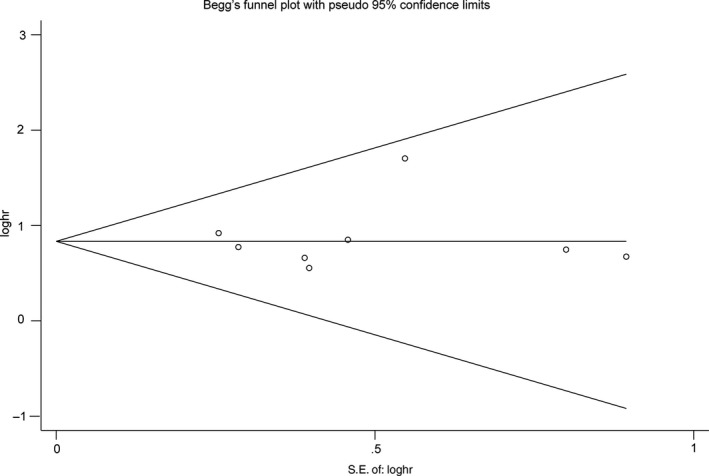
Funnel plot of the publication bias for the analysis of the pooled HRs of OS

No obvious bias was detected by the funnel plot (Figure 6) and Begg's test in HRs for OS (*P* = 1.000). We did not proceed Begg's test for other clinical parameters, because of no enough studies included (n < 10).

### 3.6 Sensitivity analysis

**Figure 7:**
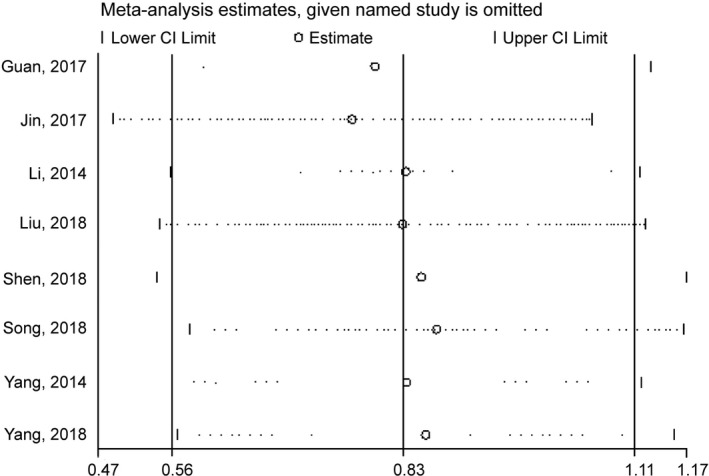
Sensitivity analysis of the pooled HRs of lncRNA GHET1 expression and OS

Sensitivity analysis was performed by removing one study every time from the pooled analysis to check the stability of results for OS of each study (Figure 7).

## 4 DISCUSSION

As lncRNAs play important roles in the process of genes’ transcription and translation, their dysregulation has been increasingly identified as a hallmark feature in the progression of cancer. GHET1, a novel identified lncRNA, was significantly over‐expressed in the gastric cancer.^36^ It has been confirmed to be an oncogene through some biological process in a variety of cancers although the role of GHET1 in cancers remains unclear yet.^17^, ^21^, ^31^, ^35^ In addition, the prognosis value of GHET1 expression in cancer and association between high expression of GHET1 and tumour stage, differentiation, has also been widely reported.^21^, ^22^, ^30^

In our meta‐analysis, we found that lncRNA GHET1 might be an unfavourable prognosis factor for cancer patients. Higher expression of GHET1 was associated with poorer OS (HR = 2.30, 95% CI: 1.75‐3.02), and the subgroup meta‐analysis stratified by analysis type, cancer type, sample size and follow‐up time showed the similar results. Furthermore, no heterogeneity was found in the association between GHET1 expression and OS. In addition, we explored the relationship between GHET1 expression and clinical parameters. We found that there was a significant positive correlation between higher GHET1 expression level and more advanced clinical stage (OR = 3.23, 95% CI: 2.25‐4.64), earlier lymph node metastasis (OR = 3.19, 95% CI: 1.85‐5.48), earlier distant metastasis (OR = 4.65, 95% CI: 1.99‐10.83) and bigger tumour size (OR = 2.95, 95% CI: 1.78‐4.89). Collectively, lncRNA GHET1 participates in the development and progression of tumours and may serve as a promising biomarker for prognosis in Asian with cancers.

A few highlights exist in our study. Firstly, this is the first study comprehensively explored the relationship between the GHET1 expression level and prognostic outcomes in human cancers based on our knowledge. Secondly, the methodology and results are credible as we followed recommendations of the PRISMA statement.^23^ Furthermore, the results are relatively accurate because of a fixed‐effect model used in most of our analysis.

However, there are some limitations in our meta‐analysis. First, the number of patients included was relatively small, which might lead to inadequate stringency. Second, all studies included were retrospective studies. Most of them were from China, which may be more preferential in China. Third, the cut‐off values for positive GHET1 expression were not always consistent in different studies. However, all of them can reflect the tendency of GHET1 expression and no statistical heterogeneity was found among them, including publication bias analysis and sensitivity analysis. Fourth, the HRs and 95% CIs of several studies were extracted from Kaplan‐Meier curves, which might affect the accuracy of consequences.

In conclusion, the higher lncRNA GHET1 expression was associated with more advanced clinical stage, earlier lymph node metastasis, earlier distant metastasis, bigger tumour size and poorer OS in cancer patients. Our meta‐analysis suggested that lncRNA GHET1 may serve as a promising biomarker for prognosis in Asian with cancers. In the future, multicentre, larger and higher‐quality studies using single standard for determining GHET1 expression are needed to identify the results of this study.

## CONFLICT OF INTEREST

The authors confirm that there are no conflicts of interest.

## AUTHOR CONTRIBUTION

Study concept and design: Yu Liu, Yao‐Fei Jiang; Bibliographic search, extraction of data: Yao‐Fei Jiang, Hong‐Yan Zhang, Hui Shen; Analysis and interpretation of the data: Jin Ke, Hai‐Bin Ou; Drafting of the manuscript: Yao‐Fei Jiang, Yu Liu, Hong‐Yan Zhang, Jin Ke; Revision of the article: Hai‐Bin Ou, Hui Shen.
